# Supplementary material for: Comparative analysis of VMAT plans on Halcyon and infinity for lung cancer radiotherapy
Source: PLoS One. 2025 Feb 11;20(2):e0318462. doi: 10.1371/journal.pone.0318462 (PMC11813144; doi:10.1371/journal.pone.0318462)
Supplement: Attachment — Submitted filename: Response to Reviewers.docx [file pone.0318462.s002.docx]

ONE-D-24-42939

Comparative Analysis of VMAT Plans on Halcyon and Infinity for Lung Cancer Radiotherapy
PLOS ONE

## Responses to Editor:

**Editor Comment 1**:

Please include the following items when submitting your revised manuscript:

- A rebuttal letter that responds to each point raised by the academic editor and reviewer(s). You should upload this letter as a separate file labeled 'Response to Reviewers'.
- A marked-up copy of your manuscript that highlights changes made to the original version. You should upload this as a separate file labeled 'Revised Manuscript with Track Changes'.
- An unmarked version of your revised paper without tracked changes. You should upload this as a separate file labeled 'Manuscript'.

**Reply 1**:

The resubmitted manuscript includes: a response to reviewers (Response_to_Reviewers.docx), a marked-up version of the manuscript (Revised_Track.pdf, generated by LaTeX Diff), and the revised manuscript without tracked changes (infi_dose_revised.pdf, generated by LaTeX source). Additionally, the anonymized raw data used for the study results, which have not been statistically processed, is uploaded as supplementary information in the file "dataset_collections.zip" in the supplement files.

**Editor Comment 2**:

If applicable, we recommend that you deposit your laboratory protocols in protocols.io to enhance the reproducibility of your results. Protocols.io assigns your protocol its own identifier (DOI) so that it can be cited independently in the future. For instructions see: [https://journals.plos.org/plosone/s/submission-guidelines#loc-laboratory-protocols](https://track.editorialmanager.com/CL0/https:%2F%2Fjournals.plos.org%2Fplosone%2Fs%2Fsubmission-guidelines%23loc-laboratory-protocols/1/010f019390052c76-2f5d6844-f175-46e9-bb34-83efac23f06b-000000/WZiTDj7bv9FG3r-L-JlcTXNbY_h963LqjcIvVjs66jc=188). Additionally, PLOS ONE offers an option for publishing peer-reviewed Lab Protocol articles, which describe protocols hosted on protocols.io. Read more information on sharing protocols at [https://plos.org/protocols?utm_medium=editorial-email&utm_source=authorletters&utm_campaign=protocols](https://track.editorialmanager.com/CL0/https:%2F%2Fplos.org%2Fprotocols%3Futm_medium=editorial-email%26utm_source=authorletters%26utm_campaign=protocols/1/010f019390052c76-2f5d6844-f175-46e9-bb34-83efac23f06b-000000/qOKzMmhymisG9vKvmPzm9tX2CMMKSfcTw0GPuVXXlG0=188).

**Reply 2**:

We have published the study protocol for this manuscript on protocols.io at the following link: http://dx.doi.org/10.17504/protocols.io.ewov1dqrkvr2/v1, and this information has been added to the Materials and Methods section of the revised manuscript.

**Editor Comment 3**:

Thank you for stating the following financial disclosure:

This research was supported by Zhejiang Province Natural Science Foundation of China under Grant No. LGF21H180014, the Medical and Health Research Project of Zhejiang Province under Grant No.2021PY002, and Zhejiang Provincial Basic Public Welfare Research Project (No. LGF22H160070).

Please state what role the funders took in the study.  If the funders had no role, please state: ""The funders had no role in study design, data collection and analysis, decision to publish, or preparation of the manuscript.""

If this statement is not correct you must amend it as needed.

Please include this amended Role of Funder statement in your cover letter; we will change the online submission form on your behalf.

**Reply 3**:

The funders had no role in study design, data collection and analysis, decision to publish, or preparation of the manuscript. We have included this statement in the revised cover letter:

 ”*This research was supported by Zhejiang Province Natural Science Foundation of China under Grant No. LGF21H180014 (Lingyun Qiu, Yucheng Li and Weijun Chen), the Medical and Health Research Project of Zhejiang Province under Grant No.2021PY002 (Kainan Shao, Lingyun Qiu, Yucheng Li and Weijun Chen), and Zhejiang Provincial Basic Public Welfare Research Project (No. LGF22H160070) (Kainan Shao and Fenglei Du). The funders had no role in study design, data collection and analysis, decision to publish, or preparation of the manuscript.*”

**Editor Comment 4**：

Your ethics statement should only appear in the Methods section of your manuscript. If your ethics statement is written in any section besides the Methods, please move it to the Methods section and delete it from any other section. Please ensure that your ethics statement is included in your manuscript, as the ethics statement entered into the online submission form will not be published alongside your manuscript.

**Reply 4**:

The ethics statement is now outlined in the Methods section of the manuscript, and the ethics statement has been removed from other sections of the manuscript.

**Editor Comment 5**:

All PLOS journals now require all data underlying the findings described in their manuscript to be freely available to other researchers, either 1. In a public repository, 2. Within the manuscript itself, or 3. Uploaded as supplementary information.

This policy applies to all data except where public deposition would breach compliance with the protocol approved by your research ethics board. If your data cannot be made publicly available for ethical or legal reasons (e.g., public availability would compromise patient privacy), please explain your reasons on resubmission and your exemption request will be escalated for approval.

The datasets generated and analyzed during the current study are not publicly available due to institutional data protection law and confidentiality of patient data but are available from the corresponding author on reasonable request in person.

**Reply 5**:

In the revised version submitted, the anonymized raw data used for the study results, which have not been statistically processed, is uploaded as supplementary information in the file "dataset_collections.zip."

**Editor Comment 6:**

6. Please include captions for your Supporting Information files at the end of your manuscript, and update any in-text citations to match accordingly. Please see our Supporting Information guidelines for more information: <http://journals.plos.org/plosone/s/supporting-information>.

**Reply 6**:

The captions of the supplementary files have been added in the Supplementary Information section of the revised manuscript.

**Editor Comment 7:**

While revising your submission, please upload your figure files to the Preflight Analysis and Conversion Engine (PACE) digital diagnostic tool, [https://pacev2.apexcovantage.com/](https://track.editorialmanager.com/CL0/https:%2F%2Fpacev2.apexcovantage.com%2F/1/010f019390052c76-2f5d6844-f175-46e9-bb34-83efac23f06b-000000/G70xV60n-iVdrl6XhK5308eyDI7Fe6M4BVMODXA1qAE=188). PACE helps ensure that figures meet PLOS requirements. To use PACE, you must first register as a user. Registration is free. Then, login and navigate to the UPLOAD tab, where you will find detailed instructions on how to use the tool. If you encounter any issues or have any questions when using PACE, please email PLOS at [figures@plos.org](mailto:figures@plos.org). Please note that Supporting Information files do not need this step.

**Reply 7**:

Images have been reviewed and checked on the (PACE) website to ensure they meet the required standards. EPS images have been used in LaTeX manuscript file, and TIF images (converted by PACE) have been uploaded separately.

## Reviewer #1

**Reviewer #1**: “*The text complies with the standard English language and was written in a clear and objective manner. The work presents a current and relevant subject and contributes to knowledge in the area covered. The objectives are clear, but could be better defined. The Introduction is well-founded and is appropriate to the proposed theme, although the reason for selecting the two treatments under study (Halcyon and Infinity) is not clear. The research method used, however, is not clearly defined and is probably not consistent with the objective of the work. The text of the manuscript does not make it clear whether this is a clinical trial (retrospective?) or a retrospective cohort. The inclusion or selection criteria for patients are not clarified, as well as the criteria for their allocation to the treatment groups. Information on blinding is also not presented. The initial characteristics of the patients are also not presented in the results. Are the distribution of sociodemographic characteristics and the severity and characteristics of the disease similar between the groups at the beginning of the research? The conclusion is based on the research results, however the methodology is fragile.*”

We sincerely appreciate the valuable feedback from the reviewers. In response to these comments, we have revised the manuscript and added some supplementary content. Below, we provide a point-by-point response to each of the reviewers' comments.

**Comment 1:** The objectives are clear, but could be better defined.

**Response 1:** At the end of the **Introduction** section, we have added the following contents to clearly state the specific objectives of this study：

“*The primary objective of this study is to compare the dosimetric characteristics and treatment efficiency of volumetric modulated arc therapy (VMAT) plans generated on Halcyon and Infinity platforms for patients with non-small cell lung cancer (NSCLC). The main study objective is to evaluate the differences in dosimetric characteristics, target coverage, normal tissue protection, and treatment efficiency between the two accelerators, yielding scientific evidence to guide the selection and optimization of radiotherapy equipment for non-small cell lung cancer.*”

**Comment 2:** The reason for selecting the two treatments under study (Halcyon and Infinity) is not clear.

**Response 1:** Halcyon and Infinity are two widely used linear accelerator platforms with distinct design philosophies, each offering unique technical features and clinical advantages. The Halcyon accelerator, a novel "ring-type" system, while Infinity represents one of the most advanced C-arm linear accelerators currently in use. The reasons for choosing these two platforms have been supplemented in the **Introduction** section. The specific content is as follows:

“*Halcyon and Infinity are two widely used linear accelerator platforms with distinct design philosophies, each offering unique technical features and clinical advantages. The Halcyon accelerator, a novel "ring-type" system, incorporates a jaw-free design, double-stacked multileaf collimators (MLCs), and operates in flattening filter-free (FFF) mode. These features enhance treatment efficiency, improve normal tissue sparing, and simplify system maintenance \cite{Lim2019} \cite{DeRoover2019} \cite{Riley2018}. Additionally, the ring gantry design facilitates faster CBCT image acquisition and reduces the risk of collisions between the gantry and the treatment couch, thereby improving safety and workflow efficiency \cite{Cai2019} \cite{Hermida2023}. In contrast, the Infinity accelerator features a traditional C-arm gantry design, a standard configuration that has been employed in clinical practice for over 30 years. Infinity is equipped with the Agility system, which incorporates multileaf collimators with finer leaf width and supports multienergy beams, providing greater flexibility in field selection and beam modulation \cite{Gholampourkashi2019}. Infinity represents one of the most advanced C-arm linear accelerators currently in use, offering well-established performance, robustness, and a larger maximum field size, which allows for more complex and adaptable treatment plans.*

*While previous studies have explored the use of VMAT technology in the treatment of other tumor sites \cite{Teoh2011} \cite{Macchia2017} \cite{Bhatt2018} \cite{Hunte2022}, there is a lack of direct clinical evidence comparing the dosimetric performance and treatment efficiency of the Halcyon and Infinity accelerators, specifically in NSCLC radiotherapy. The direct machine parameter optimization (DMPO) algorithm of the RayStation planning system \cite{Bodensteiner2018} has been shown to increase the efficiency and precision of radiotherapy plan design \cite{haardemark2003direct} \cite{Dobler2007} \cite{Dobler2009} and is widely used for generating treatment plans on both the Halcyon and Infinity platforms \cite{Saini2021}. However, the relative advantages and limitations of these two accelerators in clinical practice remain unclear. The rationale for comparing these two platforms lies in their complementary characteristics. While Halcyon emphasizes treatment efficiency, compact design, and cost reduction, Infinity focuses on versatility, large field size, and flexibility in energy selection. By directly comparing these two systems, this study aims to provide insights into their relative strengths and limitations in the context of NSCLC radiotherapy. This comparison is clinically relevant as it offers guidance on equipment selection, treatment planning strategy, and optimization of clinical workflow, thereby supporting evidence-based decision-making for clinicians and healthcare institutions.*”

**Comment 3:** The text of the manuscript does not make it clear whether this is a clinical trial (retrospective?) or a retrospective cohort. The inclusion or selection criteria for patients are not clarified, as well as the criteria for their allocation to the treatment groups. Information on blinding is also not presented.

**Response 3:** We have supplemented and clarified the study type, patient selection criteria, allocation method, and applicability of blinding in the Abstract and Materials and Methods sections. The specific adjustments are as follows:

(1) Clarification of Study Type

The following statement has been added to the **beginning** of **Materials and Methods** section, to explicitly state that this study is a retrospective cohort study:

“*This study is a retrospective cohort study involving 60 patients with non-small cell lung cancer (NSCLC) who underwent volumetric modulated arc therapy (VMAT) using Halcyon and Infinity platforms.*”

In the **Abstract**, the following statement has been added:

“*This retrospective cohort study aims to compare the dosimetric characteristics and treatment efficiency of VMAT plans delivered using Halcyon and Infinity linear accelerator platforms in patients with NSCLC.*”

(2) Inclusion and Exclusion Criteria

The following statement about the patient inclusion and exclusion criteria has been added to the **beginning** of **Materials and Methods** section:

“*Patients were included in the study if they had pathologically confirmed NSCLC, underwent routine radiotherapy during 2021-2023, with a prescription dose of 60 Gy in 30 fractions, and had complete CT image data available for treatment planning. Exclusion criteria included patients with prior thoracic radiotherapy, patients who did not receive a prescription dose of 60 Gy in 30 fractions, or those with incomplete treatment plan records.*”

(3) Patient Allocation Method

In the **Materials and Methods** section, we have clarified the patient allocation method. The following statement has been added to **line 137** of the manuscript:

“*For each patient, CT images were used to create simulated treatment plans on both the Halcyon and Infinity platforms. Patients were not directly assigned to treatment groups, as all patients underwent virtual treatment planning on both platforms to ensure consistent and comparable data.*”

(4) Applicability and Explanation of Blinding

In the **Materials and Methods** section, we have explicitly stated that blinding was not used in this study. The following statement has been added to **line 142** of the manuscript:

“*This study did not involve blinding, as all treatment plans were generated retrospectively on the same set of CT images, and dosimetric analysis was conducted using automated software with no direct human intervention during dose evaluation. Additionally, since the study used anonymized retrospective data and no direct patient intervention was involved, blinding was not applicable in this context.*”

Through these revisions and clarifications, we have more clearly defined the study type, patient selection criteria, patient allocation method, and applicability of blinding. We hope these changes address the reviewers' concerns. If there are any further suggestions or comments, we would be grateful for the opportunity to make additional improvements.

**Comment 4:** The initial characteristics of the patients are also not presented in the results.

**Response 4:** We sincerely appreciate the valuable feedback from the reviewers. Based on your suggestion, we have added **a new subsection** (Patient characteristics) at the **beginning** of the **Results** section to describe the initial characteristics of the patients, and we have also included an additional figure (**Fig. 1**) to describe the distribution of volumes of tumor targets. The specific adjustments are as follows:

“*Among the 60 enrolled patients, 32 cases (53.3%) were classified as Stage 3 (locally advanced), and 27 cases (45%) were classified as Stage 4 (metastatic disease). The distribution of tumor locations was as follows: 15 cases (25%) in the Left Upper (LU) lung, 27 cases (45%) in the Right Upper (RU) lung, 5 cases (8.3%) in the Right Middle (RM) lung, 4 cases (6.7%) in the Left Lower (LD) lung, and 9 cases (15%) in the Right Lower (RD) lung. All tumors were classified as central-type tumors. Since early-stage patients are more likely to undergo surgery or stereotactic body radiotherapy (SBRT) with large fractional doses, most of the enrolled patients were in advanced stages (Stage 3 or Stage 4), which typically require conventionally fractionated radiotherapy (60 Gy in 30 fractions). Consequently, the target volume for these patients often included the position of the cervical lymph nodes. The mean clinical target volume (CTV) was 169.75 ± 123.43 cm³, and the mean planning target volume (PTV) was 285.4 ± 161.56 cm³. Figure 1 illustrates the distribution of CTV and PTV volumes (unit: cm³) among all enrolled patients. The horizontal axis represents the volume (cm³), while the vertical axis represents the number of patients. The two curves in the figure correspond to the volume distributions of CTV and PTV, respectively. More detailed patient characteristics are provided in the Supplementary Information.*”

Through these additions, we aim to provide a more comprehensive and detailed presentation of the patient baseline characteristics, offering enhanced scientific and data-driven support for the study’s findings. We would be grateful for any further suggestions or feedback.

**Comment 5**：The conclusion is based on the research results, however the methodology is fragile.

**Response 5:** We sincerely appreciate the valuable feedback from the reviewer. In response to your comments regarding the limitations of the study methods and robustness, we have made the following adjustments in the **Discussion** section:

We have added a detailed explanation of the limitations of our study (**line 321**) :

“*Despite offering valuable insights into the dosimetric differences between the two systems, this study has several limitations. The single-center retrospective design and relatively small sample size of 60 patients may limit the generalizability of the results. Additionally, the study is confined to lung cancer patients receiving a prescription dose of 60 Gy in 30 fractions, and findings may not extend to other tumor sites or fractionation schemes. Moreover, the use of a single planning system (RayStation) introduces potential system-related biases, and the comparison focused solely on Halcyon and Infinity, without encompassing other accelerators within the C-arm category.*”

We have provided an expanded discussion on the robustness of our methodology (**line 330**) :

“*Nevertheless, we ensured the methodological robustness of this study by using a standardized treatment planning system (RayStation 9A) and consistent dose constraints, ensuring that the treatment plans for Halcyon and Infinity were generated under equivalent optimization conditions. This approach minimized potential sources of bias and enhanced the reliability of the results. The inclusion of advanced-stage NSCLC patients with a prescription dose of 60 Gy in 30 fractions presented a challenging scenario due to diverse tumor locations and large target volumes, providing a meaningful comparison of the two accelerators' clinical performance and highlighting Halcyon's potential advantages in optimizing treatment efficiency and protecting normal tissues while maintaining target coverage.*”

Through these additions, we believe we have more clearly conveyed the study's limitations and the robustness of the methods used, while also offering scientific recommendations for future research. We would greatly appreciate any further suggestions or modifications.

Once again, we sincerely thank the reviewer for their valuable feedback on this study.

## Reviewer #2

**Reviewer #2**: *I have read the article entitled: “Comparative Analysis of VMAT Plans on Halcyon and Infinity for Lung Cancer Radiotherapy “ which is about a retrospective study comparing two linear accelerators ( and Infinity) used for VMAT in NSCLC, in terms of target dose coverage, normal tissue protection, and plan complexity. The results of the study show that the Halcyon system offers enhanced protection for normal tissues and performs well in low-dose regions, while the Infinity system provides greater treatment efficiency and better control in high-dose areas. Although the research methodology appears generally sound, there are some concerns that need to be addressed:*

**Comment 1**：Confounding Factors: The study does not mention case matching by stage, tumor size, or tumor location, which could introduce variability in dose requirements across cases. This could be a significant confounding factor impacting dose comparisons. The authors could consider using regression models to account for variability, which would strengthen the analysis by reducing biases due to these unaccounted differences in patient characteristics.

**Response 1:** We sincerely appreciate the reviewer’s valuable feedback. To minimize the impact of patient characteristic differences on the dosimetric comparison, we have included the inclusion and exclusion criteria as well as a more detailed description of the patient characteristics in the revised manuscript:

(1) Inclusion and Exclusion Criteria (**line 64**):

“*Patients were included in the study if they had pathologically confirmed NSCLC, underwent routine radiotherapy during 2021-2023, with a prescription dose of 60 Gy in 30 fractions, and had complete CT image data available for treatment planning. Exclusion criteria included patients with prior thoracic radiotherapy, patients who did not receive a prescription dose of 60 Gy in 30 fractions, or those with incomplete treatment plan records.*”

(2) Patient characteristics:

At the beginning of the **Results** section (**line 177**), we have added a detailed description of the patient characteristics. Among the 60 patients enrolled, 32 (53.3%) were classified as Stage 3 (locally advanced) and 27 (45%) as Stage 4 (distant metastasis). The distribution of tumor locations is as follows: left upper (LU): 15 cases, right upper (RU): 27 cases, right middle (RM): 5 cases, left lower (LD): 4 cases, and right lower (RD): 9 cases. Given that most of the patients were in the later stages (early-stage patients are more likely to opt for surgery or stereotactic radiotherapy with high-dose fractionation instead of 60 Gy in 30 fractions), the tumor target volumes generally included the cervical lymph node areas. The description of tumor location distribution above is based on the primary tumor volume in each region. A typical diagram of the tumor target location is shown below (the green-shaded area represents the PTV):

(3) Standardization of Study Design:

The radiotherapy prescription for all patients enrolled in the study was 60 Gy in 30 fractions (conventional fractionation). Due to the advanced stages and large tumor volumes of the patients, the treatment plan was designed to ensure adequate target dose coverage (with 95% of the PTV receiving 60 Gy) while also prioritizing the protection of critical organs, particularly the normal lung tissue, to minimize the risk of radiation pneumonitis.

（4）Regarding the Regression Model:

Thanks to the reviewer for the insightful suggestions. To address the potential impact of confounding factors, we calculated the difference in dose metrics (Halcyon - Infinity) and analyzed the correlations with PTV volume (PTV_Volume), patient stage (Stage), and tumor location (Location):

| Correlation coeficient | PTV_Volume | PTV_Volume_p | Stage | Stage_p | Location | Location_p |
| --- | --- | --- | --- | --- | --- | --- |
| Lungs_Dmean_diff | 0.4807 | 0.0001 | 0.3607 | 0.0046 | 0.1191 | n.s. |
| Lungs_V5Gy_diff | 0.4151 | 0.001 | 0.2059 | n.s. | 0.0693 | n.s. |
| Heart_Dmean_diff | 0.3098 | 0.016 | 0.0209 | n.s. | 0.3137 | 0.0147 |
| SpinalCord_D0.03cc_diff | 0.2044 | n.s. | 0.1971 | n.s. | 0.2715 | 0.0359 |
| plan_mus_diff | 0.3209 | 0.0124 | -0.0243 | n.s. | 0.112 | n.s. |
| complexity_diff | 0.3816 | 0.0026 | -0.0187 | n.s. | 0.0551 | n.s. |

We have included the relevant correlation analysis in the **Results** section (**line 219**), as follows:

“*To account for potential confounding factors, we calculated the correlation coefficients and p-values between the dose metric differences (Halcyon - Infinity) and patient characteristics (PTV_Volume, Stage, and Location). The analysis revealed a positive correlation between difference of Lungs mean dose and volumes of PTV (r = 0.4807, p = 0.0001), indicating that lung mean dose differences are significantly influenced by target volume, as larger target volumes typically occur in more advanced stages. Stage also showed a moderate correlation with Lungs_Dmean_diff (r = 0.3607, p = 0.0046), further supporting this relationship. In contrast, the correlation with tumor location was weak and not significant. Difference of Heart mean dose and SpinalCord D0.03cc demonstrated significant correlations with tumor location (r = 0.3137, p = 0.0147; r = 0.2715, p = 0.0359, respectively), which can be attributed to proximity between the target volume and these critical organs.*”

In the **Discussion** section (**line 313**), we have added the following paragraph to elaborate on the significance of our findings:

“*Our analysis of potential confounding factors revealed that the differences in lung mean dose were significantly correlated with PTV volume and moderately correlated with stage, likely due to larger target volumes observed in advanced-stage NSCLC patients. In contrast, patient stage and tumor location showed weaker correlations, suggesting that these characteristics had a limited impact on the observed dose differences. These findings underscore the importance of controlling for target volume variability in future studies and considering regression models to further account for potential confounding factors.*”

This study was designed to perform a paired comparison of radiotherapy plans using the same CT images and target volumes on both the Halcyon ring accelerator and the traditional C-arm accelerator Infinity. This design minimizes inter-patient variability and provides a standardized platform for comparison, the following statements have also added to the **Discussion** section (**line 334**):

“*The inclusion of advanced-stage NSCLC patients with a prescription dose of 60 Gy in 30 fractions presented a challenging scenario due to diverse tumor locations and large target volumes, providing a meaningful comparison of the two accelerators' clinical performance and highlighting Halcyon's potential advantages in optimizing treatment efficiency and protecting normal tissues while maintaining target coverage.*”

With these additions, we believe that we have comprehensively addressed the reviewer’s suggestions regarding the regression analysis and have highlighted the scientific rigor of our study design and the clinical implications of the findings. We would be grateful for any additional recommendations.

**Comment 2**：Study Limitations:

Inter-Operator Variability: The skill and experience of planners and operators can significantly affect treatment planning quality, and this variability may influence the findings.

Case Selection Bias: If the study does not cover a diverse range of case types (e.g., varying tumor locations or complexities), it may not be possible to generalize the results. This selection bias could limit the applicability of the findings to broader clinical contexts.

**Response 2:**

We would like to express our gratitude for the reviewer’s insightful feedback regarding **Inter-Operator Variability** and **Case Selection Bias**. In light of these comments, we have expanded the **Discussion** section in the revised manuscript to provide further clarity on the study's limitations, as detailed below:

This study is a retrospective analysis where all treatment plans were derived from the same CT images and completed using the standardized RayStation 9A treatment planning system under consistent optimization parameters. The dosimetric analysis was performed using automated software, thereby reducing potential human intervention in the dose evaluation. As a result, the variability arising from differences in operator skill or experience is expected to have minimal influence on the study results. However, we acknowledge that in real-world clinical settings, differences in operator expertise can impact treatment plan quality, and this remains a crucial topic for further research.

The cohort of patients in this study consists of advanced-stage NSCLC patients receiving a prescription dose of 60 Gy in 30 fractions. Given that the patients had relatively large target volumes and a diverse distribution of tumor locations, this study provides a challenging clinical context for comparing accelerator performance. Nonetheless, the study is limited to lung cancer patients, and findings may not be directly applicable to other tumor types or radiotherapy fractionation schemes. Additionally, as only one treatment planning system (RayStation) was used, performance differences between planning systems were not evaluated. Furthermore, the comparison focused on Halcyon and Infinity, which may not fully represent the performance of all C-arm accelerators.

To address these limitations, we have included the following statement in the **Discussion** section (**line 321**):

“*Despite offering valuable insights into the dosimetric differences between the two systems, this study has several limitations. The single-center retrospective design and relatively small sample size of 60 patients may limit the generalizability of the results. Additionally, the study is confined to lung cancer patients receiving a prescription dose of 60 Gy in 30 fractions, and findings may not extend to other tumor sites or fractionation schemes. Moreover, the use of a single planning system (RayStation) introduces potential system-related biases, and the comparison focused solely on Halcyon and Infinity, without encompassing other accelerators within the C-arm category.*

*Nevertheless, we ensured the methodological robustness of this study by using a standardized treatment planning system (RayStation 9A) and consistent dose constraints, ensuring that the treatment plans for Halcyon and Infinity were generated under equivalent optimization conditions. This approach minimized potential sources of bias and enhanced the reliability of the results. The inclusion of advanced-stage NSCLC patients with a prescription dose of 60 Gy in 30 fractions presented a challenging scenario due to diverse tumor locations and large target volumes, providing a meaningful comparison of the two accelerators' clinical performance and highlighting Halcyon's potential advantages in optimizing treatment efficiency and protecting normal tissues while maintaining target coverage.*”

Despite these limitations, the study effectively simulated treatment planning for advanced NSCLC patients, focusing on the accelerator performance in the context of large target volumes and complex tumor locations. This provides valuable insights into the comparative performance of radiotherapy devices in a clinically challenging setting.

## Reviewer #3

Reviewer #3: *This study conducted a comparative analysis of VMAT plans on two linear accelerator platforms, Halcyon and Infinity, for non-small cell lung cancer (NSCLC) radiotherapy, aiming to provide data support for clinical equipment selection. Overall, this paper has a clear structure and relatively complete content, but there are some issues and areas for improvement in the following aspects:*

*1.Research Design and Methods*

**Comment 1**：The authors conducted a retrospective analysis of VMAT plans for NSCLC patients, which is a reasonable design. However, it lacks detailed descriptions of key information such as patient inclusion and exclusion criteria, tumor staging, and pre-treatment physical condition. It is recommended to add detailed descriptions of patient selection, inclusion and exclusion criteria, as well as specific steps and methods of the research design, to improve the reproducibility and transparency of the study.

**Response 1:** We sincerely appreciate the reviewer’s insightful comments regarding the patient selection criteria and the transparency of our study design. In response to these suggestions, we have made the following adjustments in the revised manuscript to improve clarity and reproducibility:

(1) In the **Materials and Methods** section, we have explicitly outlined the inclusion and exclusion criteria to enhance transparency and allow for better reproducibility (**line 64**):

“*Patients were included in the study if they had pathologically confirmed non-small cell lung cancer (NSCLC), underwent routine radiotherapy between 2021 and 2023, with a prescription dose of 60 Gy in 30 fractions, and had complete CT image data available for treatment planning. Exclusion criteria included patients with prior thoracic radiotherapy, those who did not receive the prescribed dose of 60 Gy in 30 fractions, or those with incomplete treatment plan records.*”

(2) A total of 60 patients were included, with 32 (53.3%) in Stage 3 (locally advanced) and 27 (45%) in Stage 4 (distant metastasis). The distribution of tumor locations was as follows: left upper (LU): 15 cases, right upper (RU): 27 cases, right middle (RM): 5 cases, left lower (LD): 4 cases, and right lower (RD): 9 cases. All patients' pre-treatment CT data were collected following a standardized procedure and anonymized. We have provided a more detailed description of the baseline patient characteristics at the start of the **Results** section (**line 177**).
(3) To ensure the study’s reproducibility, we have outlined the specific steps of the study design. This retrospective study involved using the same set of CT images for all patients to generate VMAT plans on both Halcyon and Infinity platforms. Treatment plans were created using RayStation 9A treatment planning software under equivalent optimization conditions. The study focused on comparing dosimetric characteristics, including target coverage, normal tissue sparing, and treatment complexity.
(4) We have further elaborated on the data processing procedures to enhance clarity. All CT data were collected using a standard protocol, anonymized, and imported into the RayStation 9A planning system. The VMAT plans were optimized for each platform using consistent dose constraints, ensuring uniformity across both systems. Dosimetric parameters were automatically extracted from the RayStation-generated reports for subsequent analysis.

By incorporating these clarifications, we believe we have improved the transparency and reproducibility of our study, allowing readers to better understand the study design and methodology. We welcome any further suggestions and appreciate your valuable input.

**Comment 2**：When optimizing VMAT plans, the authors mentioned the use of RayStation 9A software, but it didn’t elaborate on the specific process of data collection and processing, including data sources, collection methods, and processing steps. It is recommended to supplement specific details of data collection and processing to ensure the reliability and accuracy of the study.

**Response 2:** We sincerely thank the reviewer for their valuable suggestions regarding the insufficient description of the data collection and processing procedures. To enhance the reliability and reproducibility of our study, we have provided more detailed explanations of these processes in the revised manuscript:

(1) CT Image Collection and Importation: The CT images were obtained using a standardized collection process and were anonymized to protect patient confidentiality. These images were then imported into the RayStation 9A treatment planning system in DICOM format, which provided the basis for subsequent VMAT plan optimization. (**line 89**)
(2) Plan Optimization Method: The Direct Machine Parameter Optimization (DMPO) algorithm, combined with a progressive automatic optimization strategy, was utilized in the RayStation 9A system (**line 129**). The key optimization objectives were:

Primary Objective: Ensure sufficient dose coverage of the target volume (PTV).

Secondary Objective: Minimize the dose to organs at risk (OARs).

Tertiary Objective: Reduce the dose to regions outside the target volume, referred to as "remaining volume at risk" (RVR).

“The plans were optimized via the direct machine parameter optimization (DMPO) algorithm in the RayStation planning system, along with a progressive automatic optimization strategy. The primary objective was to ensure adequate dose coverage of the target volume for each patient. The secondary objective was to minimize the dose to normal organs, and the tertiary objective was to reduce the dose to other regions outside the target volume (remaining volume at risk, RVR).”

(3) Dose Constraints and Plan Design Consistency: Both plans for each patient (Halcyon and Infinity) were optimized using identical target delineation and dose constraints to ensure comparability. The Equivalent Uniform Dose (EUD) function was applied to achieve uniform dose distribution in the target volume and minimize dose to the OARs. (**line 134**)

By including these clarifications, we hope to provide a clearer understanding of our study's data collection and processing methods, thereby improving the reliability and reproducibility of the results. We welcome any further suggestions from the reviewer and greatly appreciate their constructive feedback.

*2.Results*

**Comment 3**：Although the authors provided some key data in the results section, it lacks in-depth analysis and interpretation of these data. For example, regarding the differences between Halcyon and Infinity in target dose, normal tissue protection, etc., the authors didn’t provide sufficient explanation and discussion. It is recommended to increase in-depth analysis and interpretation of the results.

**Response 3:**

We thank the reviewer for their insightful suggestions. To address the concern regarding the lack of in-depth analysis and interpretation in the Results section, we have added further analysis and interpretations based on the key results presented in the manuscript. The specific additions are as follows:

(1) Analysis of Target Dose Coverage:

- Differences in Target Dose Coverage: The results show no significant differences between the Halcyon and Infinity systems in terms of CTV and PTV dose coverage (e.g., CTV D98%, D2%, D95%, D50%, Dmean, and PTV D50%, Dmean, $P > 0.05$), indicating that both systems are comparable in terms of dose coverage of the target volumes.
- Minor but Statistically Significant Differences: We observed small yet statistically significant differences in PTV D98% and PTV D2%, with Halcyon showing a slight increase in D98% and a decrease in D2% ($\Delta D98% = +63.4$ cGy, $P < 0.001$; $\Delta D2% = -32$ cGy, $P = 0.048$). These results suggest that Halcyon provides better dose homogeneity (Homogeneity Index, HI) (Halcyon: 0.115 ± 0.018 vs. Infinity: 0.130 ± 0.021, $P < 0.001$). While these differences are statistically significant, their clinical significance is likely minimal, particularly when considering the clinical dose tolerance ranges.
- Conformity Index (CI): The Infinity system exhibited a slightly better PTV CI (Infinity: 0.857 ± 0.044 vs. Halcyon: 0.856 ± 0.042, $P > 0.05$), but the difference was not statistically significant. Given the small difference, it is unlikely to have a meaningful impact on clinical outcomes.

“*These results indicate that the Halcyon system provides slightly better homogeneity in target dose coverage, whereas the Infinity system shows a minor advantage in conformity index (CI).*”

(2) Comparison of Normal Tissue Protection:

- Low Dose Region Protection: The Halcyon system demonstrated better protection for normal tissues in the low-dose regions (e.g., Lungs V5Gy, V20Gy, and V30Gy), with reductions of -0.785%, -0.245%, and -0.365%, respectively ($P < 0.001$), although the absolute differences were small. The small but significant improvement in low-dose lung exposure could be important for reducing long-term radiation-induced toxicities.
- Mean Dose to Organs: The Halcyon system showed an advantage in reducing the mean dose to the lungs and heart (lung $\Delta$Dmean = -1.70%, heart $\Delta$Dmean = -1.08%, both $P < 0.05$), while the maximum dose to the spinal cord (SpinalCord D0.03cc) showed no significant difference between the two systems ($\Delta$ = -0.3564%, $P > 0.05$). This slight improvement in lung and heart dose might be clinically relevant, especially when considering the long-term risks of radiation-induced complications.

“*The Halcyon system demonstrated slightly better protection for the lungs and heart in low-dose regions, which could be attributed to its advanced MLC and gantry design.*”

(3) Plan Complexity and Treatment Efficiency:

The Halcyon system achieved lower plan complexity, as evidenced by fewer monitor units (MU) and less complex segments. Furthermore, it improved treatment efficiency by reducing treatment time. On the other hand, the Infinity system, while more flexible for complex target volume planning, may require additional time for plan optimization.

“*The Halcyon system achieved lower plan complexity and higher treatment efficiency, making it a potentially favorable option for routine clinical workflows.*”

*3.Discussion*

**Comment 4**: In the conclusion section, the authors pointed out that Halcyon and Infinity each have advantages in NSCLC radiotherapy, but it fails to clearly provide key factors and specific recommendations to consider when selecting the optimal radiotherapy equipment. It is recommended to strengthen the content of the discussion section, including exploring the clinical significance of the research results, comparisons with other studies, limitations of the study, and potential future research directions.

**Response 4:**

We thank the reviewer for their valuable suggestions. In response to the concerns regarding the insufficient content in the Discussion and Conclusion sections, we have added the following content in the revised manuscript. This includes the clinical significance of our results, comparisons with previous studies, key factors in selecting radiotherapy equipment, research limitations, and future research directions:

(1) We have added the following analysis of the clinical implications of our findings:

- Target Dose Coverage: Both Halcyon and Infinity showed comparable performance in target dose coverage; however, Halcyon exhibited a slight advantage in dose homogeneity, while Infinity demonstrated a marginal benefit in conformity index (CI).
- Normal Tissue Protection: Halcyon provided superior protection for normal tissues in low-dose regions (e.g., Lungs V5Gy and Heart Dmean), which is particularly beneficial for patients at high risk for radiation pneumonitis.
- Treatment Efficiency and Plan Complexity: Halcyon’s automated design allows for higher treatment efficiency and lower plan complexity, while Infinity provides greater flexibility in handling complex target volumes.

Related **discussions** have been added to **line 248** of the revised manuscript:

“*These findings suggest a potential, albeit small, advantage of the Halcyon system in optimizing radiotherapy efficiency and protecting normal tissues, making it well-suited for patients requiring low-dose sparing, such as those at higher risk for radiation pneumonitis, while Infinity may be better equipped for complex target geometries requiring superior conformity.*”

(2) We have added a comparison with existing literature. Our results align with previous studies indicating that Halcyon’s design (e.g., flattening filter free and dual-layer MLC) is more favorable for improving treatment efficiency and protecting low-dose regions. However, other studies have also supported the flexibility and superior high-dose region control of Infinity in complex planning. These results suggest that each system has its advantages, and the choice of system should be made based on clinical needs and patient characteristics.

(3) Key Factors in Selecting Radiotherapy Equipment:

- Patient Characteristics: Such as tumor size, location, complexity, and the patient's health status (e.g., lung function).
- Planning Objectives: Whether the focus is on sparing low-dose regions (e.g., Halcyon) or enhancing dose conformity in high-dose regions (e.g., Infinity).
- Clinical Scenarios: Routine treatments requiring high efficiency (suitable for Halcyon) versus complex target volume treatments (suitable for Infinity). Economic Factors: Including equipment acquisition costs and operational efficiency.

We have added specific recommendations for selecting radiotherapy equipment in the **Discussion** section (**line 295**):

“*When selecting radiotherapy equipment, factors such as patient-specific characteristics, planning goals (e.g., dose conformity vs. low-dose sparing), clinical workflow requirements, and economic considerations should be taken into account.*”

(4) Limitations of the Study:

Single-Center and Limited Sample Size: As this is a single-center study with only 60 patients, the sample size may limit the ability to generalize the results to a broader patient population.

Specific Fractionation Scheme: The study was limited to a single fractionation scheme (60 Gy in 30 fractions), and the applicability of other fractionation schemes or tumor types was not assessed.

Single Planning System: All treatment plans were based on the RayStation planning system, and performance differences with other planning systems were not evaluated.

Limited Comparison: The study compared only the Halcyon and Infinity systems, excluding other C-arm accelerators from the analysis.

we have added the following detailed explanations to the revised manuscript (**line 321**):

“*Despite offering valuable insights into the dosimetric differences between the two systems, this study has several limitations. The single-center retrospective design and relatively small sample size of 60 patients may limit the generalizability of the results. Additionally, the study is confined to lung cancer patients receiving a prescription dose of 60 Gy in 30 fractions, and findings may not extend to other tumor sites or fractionation schemes. Moreover, the use of a single planning system (RayStation) introduces potential system-related biases, and the comparison focused solely on Halcyon and Infinity, without encompassing other accelerators within the C-arm category.*”

(5) Future Research Directions:

- Conduct multicenter studies with larger sample sizes to improve the robustness and generalizability of the results.
- Investigate the performance of different accelerators and planning systems across various tumor types and fractionation schemes.
- Include comparisons between multiple planning systems and other accelerators to better understand system performance variability.
- Explore the potential of emerging technologies, such as AI-driven optimization algorithms, to improve radiotherapy planning and outcomes.

We have included the following directions for future research (**line 340**):

“*Future studies should aim to expand the sample size, include multiple tumor types, and adopt prospective and multicenter designs to validate the findings. Integrating data from various planning systems and accelerators could provide a more comprehensive evaluation of their clinical performance. Additionally, the combination of different planning systems and accelerators should be explored to better understand their effects on radiotherapy outcomes. As new technologies and AI-driven optimization algorithms continue to evolve, future research should focus on their integration to further enhance the quality and efficiency of radiotherapy planning, ultimately optimizing clinical treatment equipment and workflows.*”

Through these additions, we aim to provide a more thorough exploration of the clinical significance of the study, offer clearer recommendations for selecting radiotherapy equipment, and outline meaningful directions for future research. We welcome any further suggestions to enhance the manuscript.

*4.Ethics*

**Comment 5**: Although the authors mentioned the approval of ethical review, it didn’t elaborate on how patient privacy and rights are protected during the research process. It is recommended to add detailed descriptions of ethical review and compliance, including how patient privacy and rights are protected during the research process.

**Response 5:**

We sincerely thank the reviewer for their valuable feedback. In response to your suggestions, we have made detailed modifications and additions to the Materials and Methods section to more effectively communicate the retrospective nature of the study, while also providing additional information regarding ethical review and patient privacy measures.

In the Simulation and Positioning section, we have explicitly stated that the CT images were collected as part of the patients' routine radiotherapy treatment, rather than being newly obtained for this study. The imaging data were acquired using the Brilliance Big Bore CT simulation system (Philips, Netherlands) during standard clinical practice. All images were anonymized at the time of collection and were then imported in DICOM format into the RayStation 9A planning system for the simulation treatment plans used in this study.

In the Delineation of Target Volumes and Organs at Risk section, we have revised the description to clarify that target volumes and organs at risk (OAR) were delineated by experienced radiation oncologists during the routine clinical treatment process, using existing clinical data. No new delineation of target volumes or OARs was performed for this study.

This study was approved by the Medical Ethics Committee of Zhejiang Provincial People's Hospital (Approval No. QT2024085) and fully complied with the principles of the Helsinki Declaration and relevant ethical regulations. Given that the patient data used were anonymized imaging data and no direct treatment interventions were performed, the Medical Ethics Committee granted a waiver of informed consent for this study.

With these revisions, we believe the retrospective nature of this study and the measures taken to protect patient privacy are now more clearly outlined. Should you have any further suggestions, we would be grateful for your feedback.

## Reviewer #4

**Reviewer #4:** *This study provides a coherent and useful summary of a study that compares two linear accelerator platforms for the treatment of NSCLC. The authors have explicitly articulated their purpose, methodology, principal findings, and conclusions.*

*Rationale for Acceptance:
The comparison of Halcyon and Infinity platforms for NSCLC treatment is pertinent to radiation oncology, offering critical insights for doctors in equipment selection.*

*The methodology is robust, featuring an adequate sample size and a well-defined planning and review process. Employing uniform dose restrictions and optimization parameters enhances the credibility of the comparison.*

*Notable Discoveries: The findings underscore significant disparities between the two systems, especially with normal tissue preservation and high-dose management. These findings possess significant ramifications for treatment planning and patient outcomes.*

*The research presented unequivocally substantiates the conclusions, offering significant information for practitioners. The authors duly recognize the constraints of their work and provide avenues for subsequent investigation.*

*This abstract effectively advocates for the publishing of the complete manuscript. The study looks into an important clinical question, uses a solid method, and comes up with interesting results that could change the way radiotherapy is done in the future..*

**Comment 1**: Nonetheless, I recommend that the authors contemplate a little expansion of the "plan complexity" component in the study. Although they reference a reduced number of monitor units for Halcyon, a quick mention of additional complexity metrics, such as the modulation complexity score, would enhance their argument.
**Response 1:**

Thank you for your valuable comments. As plan complexity is a crucial factor in evaluating radiotherapy plans, we have expanded the discussion on complexity metrics to further strengthen the argument of our study.

(1) Beyond the reduction in total monitor units (MU) discussed previously, we have introduced the Modulation Complexity Score (MCS) as an additional complexity metric. The MCS is defined as follows:

where $MU_{tot}$ is the total number of monitor units for the field, i denotes the number of control points, $MU_i$ represents the number of monitor units for the beam aperture at the i-th control point, AP represents the perimeter of the beam aperture, and AA represents the area of the beam aperture. A higher MCS indicates greater plan complexity.

In this study, the Halcyon system, with its uniformly set control point interval of 2° gantry rotation and its dual-layer MLC design, results in simpler aperture shapes at each control point, thereby exhibiting lower plan complexity.

(2) The reduced complexity of the Halcyon system not only simplifies plan optimization and execution but also improves the treatment delivery pass rate—meaning the difference between the planned and delivered doses is minimized. This is particularly important for ensuring precise and accurate treatment, contributing to the overall reliability of the dose distribution. Additionally, the reduction in monitor units and complexity leads to shorter treatment times, enhancing the overall efficiency of clinical workflows.

(3) We have added the following content to the **Discussion** section (**line 304**) :

“*In addition to the reduced number of monitor units, the Halcyon system demonstrated lower plan complexity, as reflected by its modulation complexity score (MCS). The simplicity of beam apertures and fewer total monitor units contributed to this advantage. Lower plan complexity not only improves treatment delivery accuracy, reducing the difference between planned and delivered doses, but also enhances clinical workflow efficiency.*”

These additions, we believe, will help further reinforce the argument concerning plan complexity in this study. We would greatly appreciate any further suggestions you might have. Once again, we thank the reviewer for their insightful guidance and feedback.
