# Supplementary material for: Comparative analysis of VMAT plans on Halcyon and infinity for lung cancer radiotherapy
Source: PLoS One. 2025 Feb 11;20(2):e0318462. doi: 10.1371/journal.pone.0318462 (PMC11813144; doi:10.1371/journal.pone.0318462)
Supplement: Attachment — Submitted filename: new_Response_to_Reviewers.docx [file pone.0318462.s003.docx]

PONE-D-24-42939R1

Comparative Analysis of VMAT Plans on Halcyon and Infinity for Lung Cancer Radiotherapy

**Journal Requirements:**

**Please review your reference list to ensure that it is complete and correct. If you have cited papers that have been retracted, please include the rationale for doing so in the manuscript text, or remove these references and replace them with relevant current references. Any changes to the reference list should be mentioned in the rebuttal letter that accompanies your revised manuscript. If you need to cite a retracted article, indicate the article’s retracted status in the References list and also include a citation and full reference for the retraction notice.**

**Response：**The format of the references was checked and the DOIs for some entries were supplemented.

## Reviewer #1

**Reviewer #1: After revision, the manuscript meets the criteria for publication. The text is written in a fluent and grammatically correct manner. The introduction adequately presents the topic and the knowledge gap to be addressed, while also contextualizing the relevance and justification of the research. The problem is appropriately described, as are the study objectives. The methodology employed is suitable for the objective and is clearly described. However, the analyses could benefit from incorporating classical association measures for cohort studies. The results are adequately presented and are directly related to the article's objective. In the discussion section, the authors examine the results in light of other similar studies, correctly present the research limitations, and provide suggestions for further advancements. The conclusion appropriately addresses the research objectives.**

**Response 1：**

Thank you for your positive evaluation of our manuscript and for your insightful suggestion to incorporate classical association measures for cohort studies. We are pleased to inform you that we have already implemented one of the most widely used classical association measures, Odds Ratios (ORs), in our analysis through logistic regression. This approach quantifies the associations between patient characteristics (such as PTV volume, tumor stage, and tumor location) and dose metric differences (Halcyon - Infinity), providing robust and interpretable results.

For instance, the logistic regression results in our manuscript include:

- Lungs_Dmean: PTV volume as a significant predictor (OR = 1.01, 95% CI: 1.005 - 1.015, p = 0.002);
- Heart_Dmean: Tumor location as a significant predictor (OR = 1.50, 95% CI: 1.10 - 2.00, p = 0.014);
- Plan Complexity: PTV volume as a significant predictor (OR = 1.02, 95% CI: 1.005 - 1.025, p = 0.001).

These ORs provide clear and interpretable measures of association, commonly used in cohort studies to describe the relative impact of independent variables on outcomes.

To ensure the manuscript reflects this point clearly, we have added the following clarification in the discussion section (**line 347**):

"*The use of logistic regression analyses in this study provides classical association measures, such as odds ratios, to quantify the relationships between patient characteristics and dose metric differences. This approach aligns with standard practices for assessing associations in cohort-like designs and offers a robust interpretation of the study results.*"

We hope this addresses your concern and appreciate your thoughtful feedback, which has further strengthened the clarity and rigor of our manuscript.

## Reviewer #2

**Reviewer #2:**

**I have read the revised version of the article: “Comparative Analysis of VMAT Plans on Halcyon and Infinity for Lung Cancer Radiotherapy “, and can say the following regarding the answers to my comments made on the original version.
The authors tried to answer to the comments, however, I still have a concern about the existence of confounding factors (comment 1). The authors answered by calculating the correlation coefficient between dose metrics and other factors. This calculation showed that, indeed, there may be possible confounding factors. They didn’t realise a logistic regression, but they recognise the possibility the existence of such confounding factors in the discussion. Scientifically, I think that authors should, at least, mention this in the abstract to avoid any misleading of the readers.**

**Response 2:**

We appreciate the reviewer’s insightful comment regarding the potential confounding factors and the suggestion to perform logistic regression analysis. To address this concern comprehensively, we have conducted a detailed logistic regression analysis to evaluate the relationships between dose metric differences (Halcyon - Infinity) and patient characteristics, including PTV volume, tumor stage, and tumor location. This analysis allowed us to quantify the contributions of these factors to dose discrepancies while accounting for confounding effects. The results revealed that PTV volume was consistently a significant predictor for dose metric differences, highlighting its critical role as a confounding factor. Tumor stage and tumor location showed variable effects depending on the specific dose metric being analyzed.

The key results of the logistic regression analysis are summarized in the table below:

| **Dose Metric** | **Variable** | **OR** | **95% CI** | **p-value** | **Interpretation** |
| --- | --- | --- | --- | --- | --- |
| **Lungs_Dmean** | PTV_Volume | 1.01 | 1.005 - 1.015 | 0.002 | Significant predictor; larger PTV volume associated with higher dose differences. |
|  | Stage | 1.30 | 0.70 - 2.40 | 0.38 | n.s. |
|  | Location | 0.72 | 0.40 - 1.20 | 0.12 | n.s. |
| **Heart_Dmean** | PTV_Volume | 1.02 | 0.98 - 1.06 | 0.32 | n.s. |
|  | Stage | 0.90 | 0.50 - 1.60 | 0.72 | n.s. |
|  | Location | 1.50 | 1.10 - 2.00 | 0.014 | Significant predictor; tumor location near the heart influences dose differences. |
| **SpinalCord_D0.03cc** | PTV_Volume | 1.00 | 0.97 - 1.03 | 0.88 | n.s. |
|  | Stage | 1.10 | 0.65 - 1.80 | 0.75 | n.s. |
|  | Location | 1.40 | 1.05 - 1.90 | 0.022 | Significant predictor; tumor location near the spinal cord influences dose differences. |
| **Plan MUs** | PTV_Volume | 1.01 | 1.003 - 1.017 | 0.005 | Significant predictor; larger PTV volume associated with more monitor units. |
|  | Stage | 1.20 | 0.85 - 1.80 | 0.45 | n.s. |
|  | Location | 0.80 | 0.60 - 1.20 | 0.28 | n.s. |
| **Complexity** | PTV_Volume | 1.02 | 1.005 - 1.025 | 0.001 | Significant predictor; larger PTV volume associated with increased complexity. |
|  | Stage | 1.05 | 0.90 - 1.25 | 0.65 | n.s. |
|  | Location | 0.90 | 0.75 - 1.10 | 0.48 | n.s. |

To ensure clarity and transparency, we have incorporated the following updates into the manuscript:

**Abstract:**

We have added a statement in the abstract to explicitly mention the logistic regression analysis and its key findings, ensuring readers are informed of the potential confounding factors and their impact on the study results. The updated abstract includes the following sentence:

“*The study also explores potential confounding factors that may influence treatment outcomes.*”

“*Logistic regression was performed to account for potential confounding factors, such as PTV volume, tumor stage, and tumor location.*”

"*Logistic regression analysis revealed that PTV volume was a significant predictor for dose metric differences, while tumor stage and tumor location had variable effects depending on the dose metric, highlighting the need to account for these factors in clinical comparisons.*"

**Materials and Methods (line 174):**

We have detailed the methods used for the logistic regression analysis, including the dependent and independent variables, and described the statistical approach employed to evaluate the confounding effects. Specifically, we stated:

"*Logistic regression analysis was performed to investigate the relationship between dose metric differences (Halcyon - Infinity) and patient characteristics, including PTV volume (continuous variable), tumor stage (categorical variable), and tumor location (categorical variable). Odds ratios (ORs) with 95% confidence intervals (CIs) were calculated to assess the strength of associations, with statistical significance set at p < 0.05.*"

**Results :**

The results section now includes a comprehensive summary of the logistic regression findings for each dose metric. For example, we highlighted the significant impact of PTV volume on lung mean dose differences (Lungs_Dmean, OR = 1.01, 95% CI: 1.005 - 1.015, p = 0.002) and noted the influence of tumor location on heart mean dose and spinal cord dose. Additionally, we added the following statement to emphasize the broader implications:

**(line 225)**

“*To further investigate potential confounding factors, we performed a logistic regression analysis to model the relationship between dose metric differences (Halcyon - Infinity) and various patient characteristics (PTV volume, tumor stage, and tumor location).The results revealed that PTV volume was a significant predictor for differences in lung mean dose, with an odds ratio (OR) of 1.01 (95% CI: 1.005 - 1.015, p = 0.002), indicating that larger target volumes were associated with higher deviations in lung dose between the two platforms. In contrast, neither tumor stage (OR = 1.30, 95% CI: 0.70 - 2.40, p = 0.38) nor tumor location (OR = 0.72, 95% CI: 0.40 - 1.20, p = 0.12) showed significant effects on lung mean dose differences. For heart mean dose, the analysis demonstrated that tumor location was a significant factor (OR = 1.50, 95% CI: 1.10 - 2.00, p = 0.014), reflecting the anatomical proximity of the heart to the target volume. However, PTV volume (OR = 1.02, 95% CI: 0.98 - 1.06, p = 0.32) and tumor stage (OR = 0.90, 95% CI: 0.50 - 1.60, p = 0.72) did not significantly influence heart dose differences. Similarly, for spinal cord maximum dose, tumor location had a significant impact (OR = 1.40, 95% CI: 1.05 - 1.90, p = 0.022), while PTV volume (OR = 1.00, 95% CI: 0.97 - 1.03, p = 0.88) and tumor stage (OR = 1.10, 95% CI: 0.65 - 1.80, p = 0.75) were not significant predictors.*”

**(line 248)**

"*The analysis for plan monitoring units (plan MUs) and plan complexity similarly indicated that PTV volume was a significant predictor, demonstrating its consistent impact across multiple dose and plan-related parameters.*"

**Discussion (line 327):**

In the discussion, we have expanded the interpretation of these findings, emphasizing the importance of controlling for PTV volume variability as a key confounding factor and addressing the limited impact of tumor stage and location on certain dose metrics. Specifically, we added:

"*Our logistic regression analysis provided further insight into the potential confounding factors influencing the differences in dose metrics between the two linear accelerators. The analysis revealed that PTV volume was a significant predictor of lung mean dose differences (OR = 1.01, p = 0.002), suggesting that larger target volumes are associated with greater variability in dose distribution. This finding underscores the importance of controlling for target volume variability when comparing radiotherapy platforms, as it may introduce systematic differences in dose metrics. In contrast, tumor stage and tumor location were not significant predictors of lung mean dose differences, indicating that these patient characteristics may have a limited impact on lung dose variability in this context. However, for heart mean dose and spinal cord dose, tumor location showed a statistically significant association, likely due to the anatomical proximity of these critical organs to the target volume. These results highlight the need to consider organ-specific dose metrics when evaluating the performance of different radiotherapy platforms. Additionally, we acknowledge that the inclusion of patients with varying PTV volumes and tumor locations posed challenges in the comparative analysis. While our study design, using matched CT images and target volumes for both platforms, minimized inter-patient variability, these factors may still introduce residual confounding. Future studies with larger sample sizes and prospective designs could further elucidate the relationships between patient characteristics and dose differences, particularly for critical organs at risk. The use of logistic regression analyses in this study provides classical association measures, such as odds ratios, to quantify the relationships between patient characteristics and dose metric differences. This approach aligns with standard practices for assessing associations in cohort-like designs and offers a robust interpretation of the study results. The observed impact of PTV volume is consistent with clinical expectations, as ensuring adequate prescription dose coverage to larger PTVs inherently poses greater challenges in sparing organs at risk. This makes cases with larger PTV volumes particularly valuable for evaluating and distinguishing the performance of different radiotherapy platforms.*"

By incorporating logistic regression analysis and explicitly discussing its findings in the abstract, methods, results, and discussion sections, we have ensured that the potential confounding factors are comprehensively addressed in the manuscript. We are confident that these revisions strengthen the scientific robustness of the study and provide valuable insights into the interpretation of dose metric differences between the Halcyon and Infinity platforms.

Thank you again for your constructive feedback, and we look forward to further suggestions to improve the manuscript.

## Reviewer #3

**Reviewer #3:** **(No Response)**

**Response 3**: None

## Reviewer #4

**Reviewer #4:** **This article presents a relevant comparison of two linear accelerator platforms used in radiotherapy for NSCLC. However, there are several areas that need improvement before it's ready for publication:
Strengths:
Relevant Topic: The comparison of Halcyon and Infinity is clinically relevant, as these are widely used platforms, and understanding their strengths and weaknesses can aid in treatment planning and equipment selection.
Clear Objective and Methodology: The study clearly defines its objective and methodology, making it easy to understand the study design and analysis.
Detailed Analysis: The article provides a detailed analysis of various dosimetric parameters and treatment efficiency factors.Acknowledges Limitations: The authors acknowledge the limitations of their study, including the small sample size and retrospective design. The article has the potential to be a valuable contribution to the literature. The article can now be suitable for publication.**

**Response 4**:

Thank you for your positive and constructive feedback on our manuscript. We are delighted that you found our study on the comparison of Halcyon and Infinity platforms in radiotherapy for NSCLC to be clinically relevant and a potential valuable contribution to the literature. We sincerely appreciate your recognition of the following strengths in our study:

- The clinical relevance of comparing Halcyon and Infinity platforms, which supports treatment planning and equipment selection.
- The clear definition of the study’s objective and methodology, ensuring transparency and understanding.
- The detailed analysis of dosimetric parameters and treatment efficiency, which highlights the strengths and weaknesses of each platform.
- The acknowledgment of study limitations, including the small sample size and retrospective design, to ensure balanced and realistic interpretation of the results.

We are encouraged by your comments and are confident that the study provides meaningful insights into the performance of these two widely used linear accelerator platforms. By addressing key clinical questions, we hope this research will support radiotherapy practitioners in optimizing treatment planning and decision-making.

As no further specific suggestions for improvement were raised, we have reviewed the manuscript carefully to ensure it remains clear, scientifically robust, and clinically relevant. Should you have any additional feedback, we would be happy to incorporate further revisions.

Thank you once again for your thoughtful review and your recognition of the potential impact of our study.
